# A Case of Hydroxychloroquine Toxicity as Evidenced by Visual Field Changes

**DOI:** 10.7759/cureus.53500

**Published:** 2024-02-03

**Authors:** Aliya Centner, Donald J Centner

**Affiliations:** 1 Medicine, University of Central Florida College of Medicine, Orlando, USA; 2 Ophthalmology, Magruder Eye Institute, Orlando, USA

**Keywords:** lupus erythematosus, retinal hydroxychloroquine toxicity, macula, visual field, hydroxychloroquine

## Abstract

Hydroxychloroquine sulfate (Plaquenil®) is a disease-modifying anti-rheumatic drug (DMARD) utilized in the management of autoimmune diseases. While its immunomodulatory actions offer therapeutic benefits, a rare complication, hydroxychloroquine-induced retinal toxicity, poses a significant concern. We present the case of an 83-year-old patient with cutaneous lupus undergoing periodic hydroxychloroquine screening for eight years. Visual field changes consistent with retinal toxicity were observed. Chart review revealed subtle visual field depression two years prior. This highlights the importance of vigilance toward unexplained visual changes and subtle changes on ophthalmologic examination in hydroxychloroquine-treated patients. Our findings underscore the necessity of baseline and periodic ophthalmologic examinations with particular attention paid to visual field depression or deficit that might occur without macular changes. Additionally, we address the choice between red and white visual field testing. This case contributes to the understanding of hydroxychloroquine-induced retinal toxicity, emphasizing the importance of comprehensive ophthalmologic surveillance in long-term users.

## Introduction

Hydroxychloroquine sulfate (Plaquenil®) is a disease-modifying anti-rheumatic drug (DMARD) commonly used in the treatment of autoimmune diseases, such as lupus erythematosus and rheumatoid arthritis. The specific mechanism of action of hydroxychloroquine is not fully elucidated [1]. It accumulates in lysosomes and increases the pH [2]. It has immunomodulatory actions that reduce inflammation and immune activation [3]. The anti-inflammatory effect is likely due to a decrease in cytokine production through alteration of transcription, specifically interleukins IL-6 and IL1β [4]. In patients with lupus, hydroxychloroquine therapy reduces organ damage, the risk of infection, and the risk of thrombosis [3]. It increases remission of lupus nephritis, decreases steroid use, and increases survival [3].

A rare but serious complication of hydroxychloroquine use is retinal toxicity. Hydroxychloroquine-induced retinopathy is much more likely to occur in individuals on long-term therapy [1]. Other risk factors include age over 60 years old, kidney or liver disease, or an existing retinal disease [1]. Hydroxychloroquine retinopathy is typically described as bull’s eye maculopathy caused by parafoveal mottling of the retinal pigment epithelium (RPE) [4]. On optical coherence tomography (OCT) imaging, loss of photoreceptor layers beginning in the ellipsoid zone may be seen [4]. In patients of Asian descent, the retinal toxicity may be outside of the macula initially [5]. Retinal toxicity is not reversible or treatable [6]. Hydroxychloroquine should be discontinued if retinal or visual changes are observed [5].

## Case presentation

An 83-year-old patient with a history of cutaneous lupus presented to the ophthalmology clinic for a routine eye examination and hydroxychloroquine screening. The patient reports that her vision is stable and denies any ocular complaints. The patient has been receiving hydroxychloroquine 200-300 mg daily since the diagnosis of lupus in 2014. The patient has a past medical history of hypertension treated with amlodipine 5 mg and hypothyroidism treated with levothyroxine 50 mcg. The patient has no history of renal or liver disease. Her past ocular history includes a history of cataracts.

On examination, the distance visual acuity was measured at 20/25-2 oculus dexter (OD) and 20/25+2 oculus sinister (OS).

**Figure 1:**
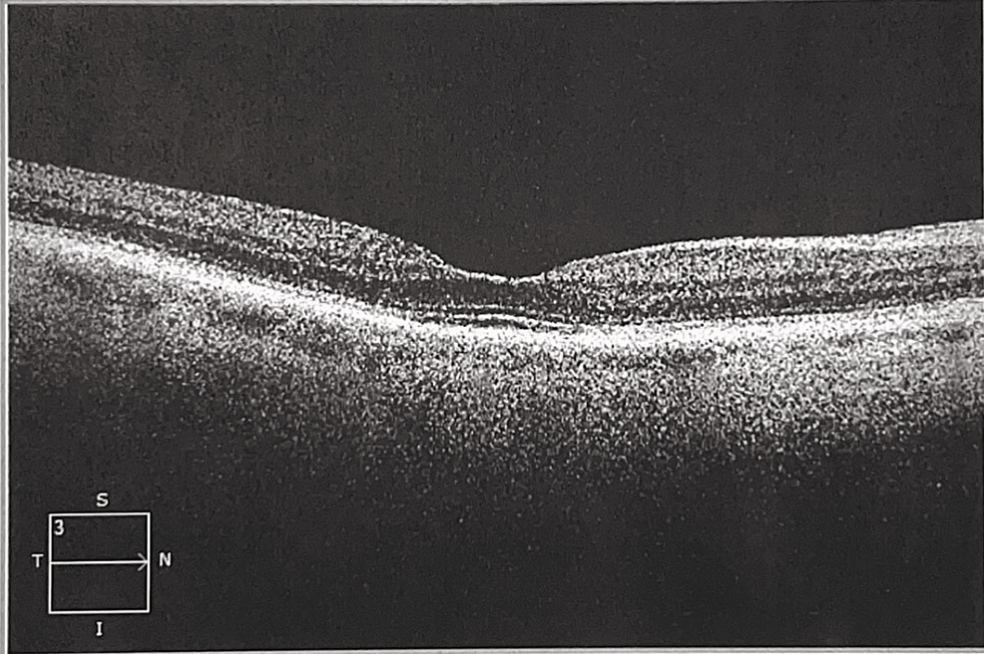
Macular optical coherence tomography OD Mac OCT oculus dexter (OD) shows no loss of photoreceptor layers.

**Figure 2:**
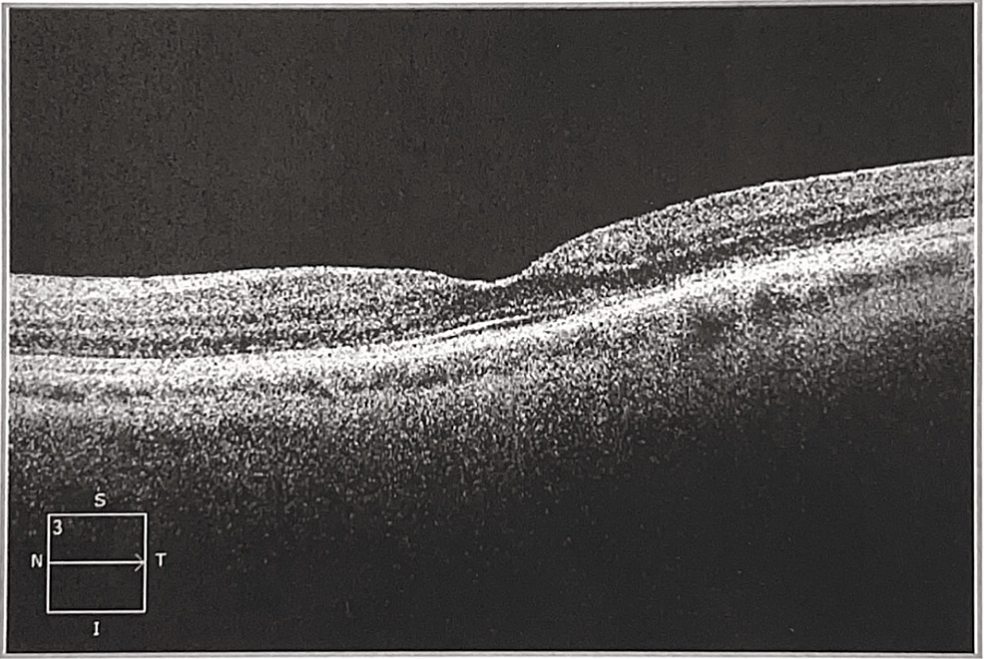
Macular optical coherence tomography OS Mac OCT oculus sinister (OS) shows no loss of photoreceptor layers.

The anterior slit lamp examination and dilated fundus examination were completed. The slit lamp examination revealed a few punctate epithelial defects on both corneas. In both eyes, a cataract (3+ nuclear sclerosis and 2+ cortical spokes) was noted, and the exam of the retina was unremarkable, without a bullseye defect in the macula. The OCT did not show any photoreceptor loss (Figures 1-2).

The Humphrey visual field (HVF) 10-2 with a red target revealed bilateral dense 360° paracentral scotoma surrounding foveal fixation (2°-7° from fixation). The patient was informed of this result and was directed to discontinue hydroxychloroquine and to follow up with rheumatology. The patient was referred to the retina clinic and was evaluated the following month. Visual acuity, slit lamp examination, dilated fundus examination, and macular OCT were unchanged. Fluorescein angiography did not reveal a bullseye defect in either macula.

Records from her previous ophthalmologist were obtained and reviewed. The patient was first evaluated in 2014, a few months after starting hydroxychloroquine 300 mg daily for the treatment of cutaneous lupus. The baseline ophthalmologic exam was unremarkable. The patient had been followed every 6-12 months since the initial visit. The patient’s medical history and exam had remained stable.

**Figure 3:**
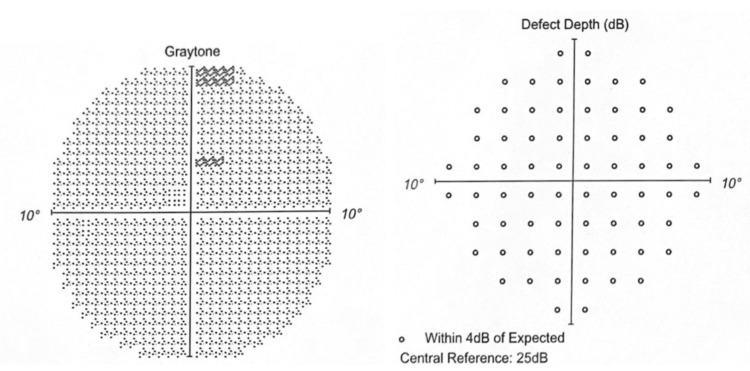
OD in 2020 Graytone OD shows two points of depression without matching depth defect.

**Figure 4:**
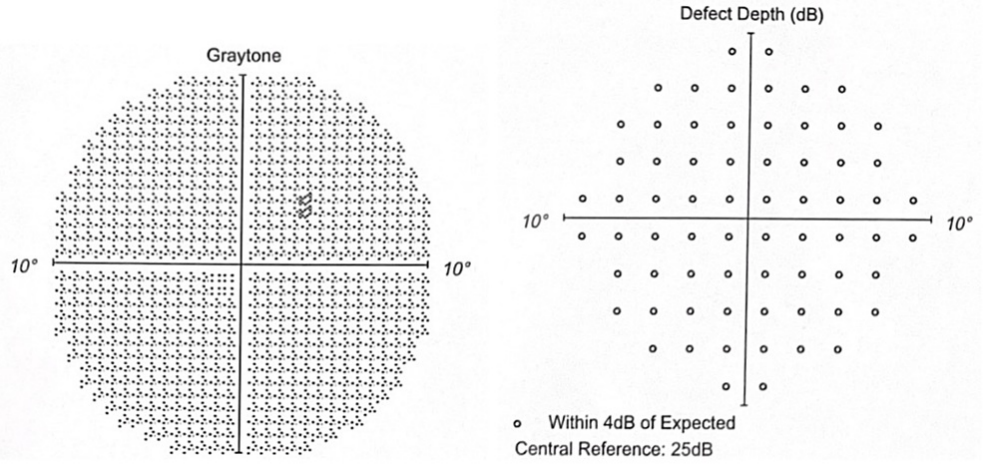
OS in 2020 Graytone OS shows one point of depression without matching depth defect.

The Humphrey visual field 10-2 (red) in 2020 showed minimal depression without any depth defect (Figures 3-4).

**Figure 5:**
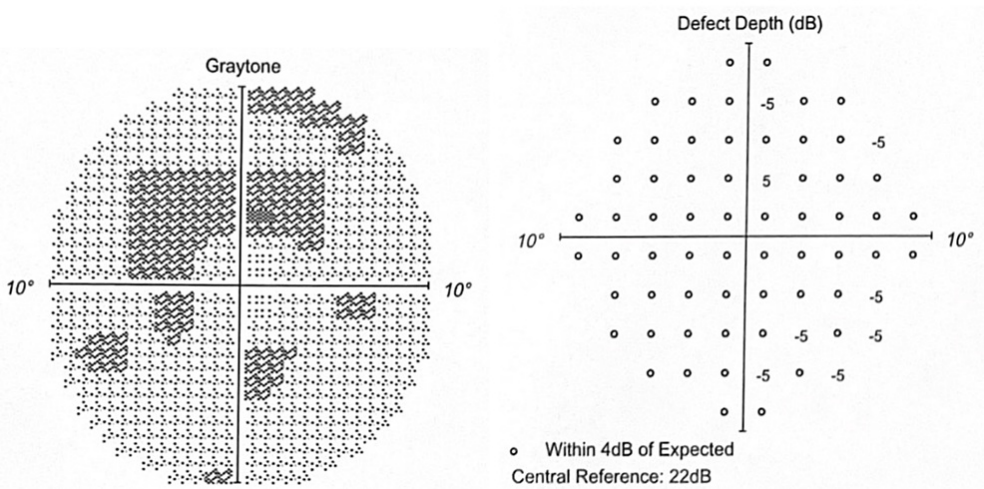
OD in 2021 Graytone OD shows early paracentral depression with minimal depth defects.

**Figure 6:**
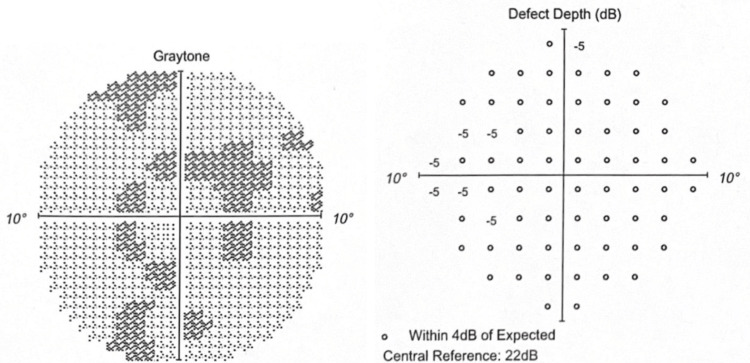
OS in 2021 Graytone OS shows paracentral depression with minimal depth defect.

The Humphrey visual field 10-2 (red) in 2021 revealed early paracentral depression with minimal depth defects (Figures 5-6).

**Figure 7:**
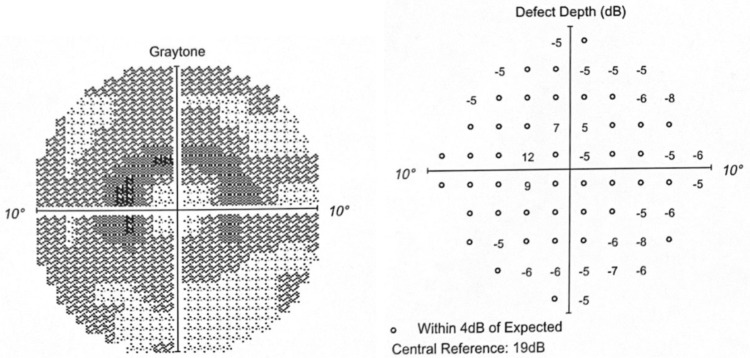
OD in 2022 Graytone OD shows graytone paracentral depression with increasing depth defects.

**Figure 8:**
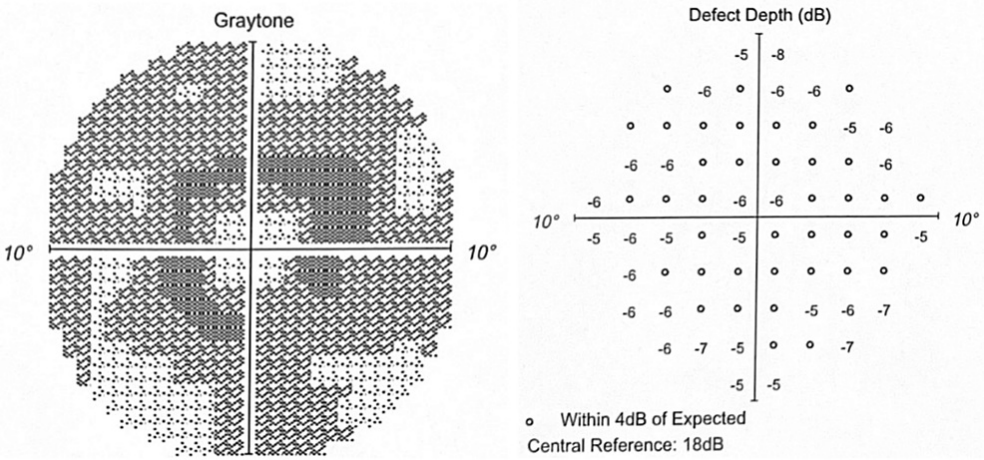
OS in 2022 Graytone OS shows graytone paracentral depression with increasing depth defects.

The Humphrey visual field 10-2 (red) in 2022 revealed worsening graytone paracentral depression with increasing depth defects (Figures 7-8).

**Figure 9:**
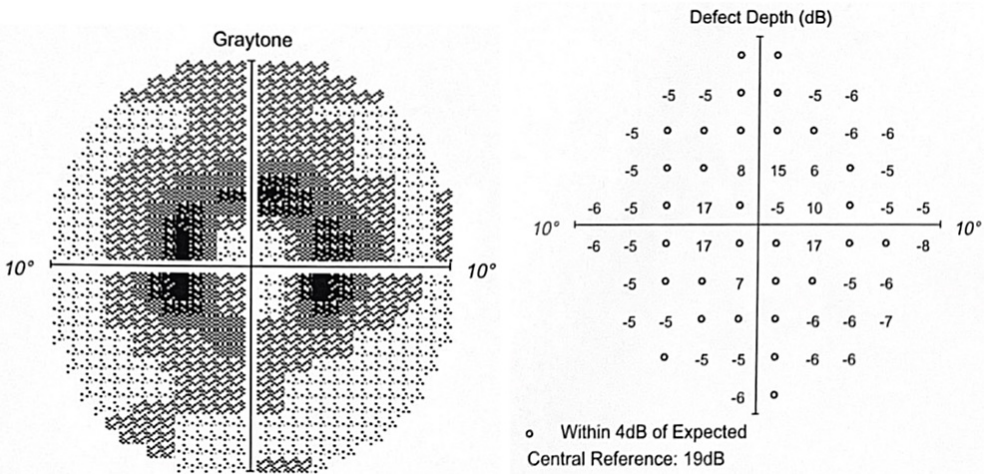
OD in 2023 Graytone OD shows dense paracentral scotoma with matching depth defects.

**Figure 10:**
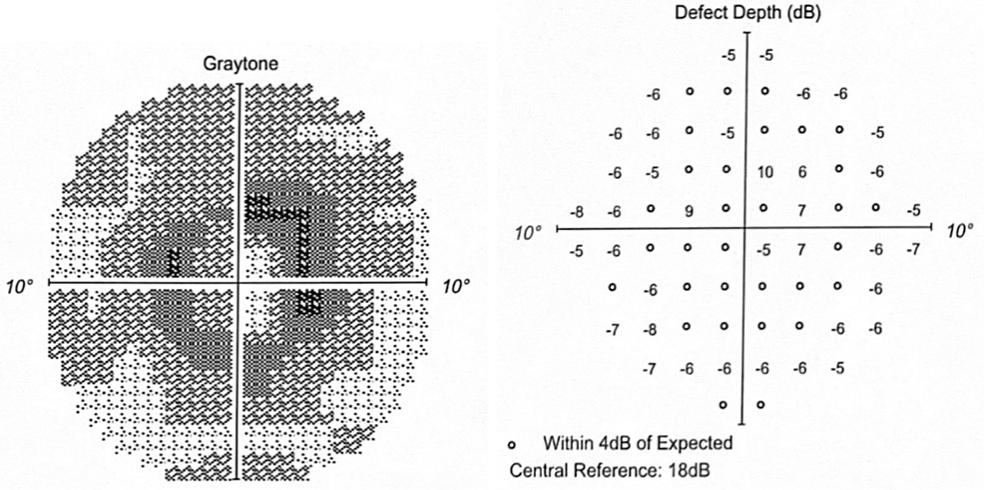
OS in 2023 Graytone OS shows dense paracentral scotoma with matching depth defects.

The paracentral scotoma on the Humphrey visual field 10-2 (Red) in 2023 had increased in graytone density and depth (Figures 9-10).

## Discussion

In this report, we present a case of hydroxychloroquine-induced retinal toxicity with a paracentral scotoma. At the time of this finding, the patient had been undergoing annual toxicity screening. It is recommended that patients beginning long-term hydroxychloroquine use should receive a baseline ophthalmologic examination within a year of the initial dose. The fundus examination of the macula is critical at the baseline examination. The exam should include visual fields and spectral-domain optical coherence tomography (SD-OCT).

Annual screening is recommended after five years on hydroxychloroquine if there were no concerns at the initial baseline screening. If there are any abnormalities noted or the patient has major risk factors, screening time can be adjusted to be more frequent.

Even though the patient did not have bullseye maculopathy, visual field change consistent with retinal toxicity was evident after nearly eight years of treatment with hydroxychloroquine. However, upon review of previous records, there was depression in her visual fields two years prior.

There have been cases of hydroxychloroquine retinopathy in which the patient has abnormal visual fields but without clear photoreceptor or RPE defects, causing the provider to overlook the retinopathy in the initial evaluation of images [4].

Another important point of consideration that arises from this case is in regard to the use of white or red visual field testing in hydroxychloroquine toxicity screening. This patient underwent red visual field testing. The American Academy of Ophthalmology recommends white 10-2 visual field testing to screen for hydroxychloroquine toxicity [7]. Red 10-2 visual field testing is often done, and studies have shown that both are acceptable [7]. Red fields have been found to be more sensitive, particularly for early signs of retinopathy, but are less specific than white fields [7]. Red fields are 91% sensitive but 57% specific, whereas white fields are 78% sensitive and 84% specific [8]. Pattern deviation plots for white fields have been found to be more consistent on repeat testing [7]. Therefore, providers could consider testing with a white target instead of a red target on the HVF 10-2.

## Conclusions

In conclusion, we report a case of hydroxychloroquine-induced retinal toxicity with a paracentral scotoma but no obvious photoreceptor or retinal pigment epithelium defects on imaging or examination. This case informs us that in hydroxychloroquine treatment, ophthalmologists need to be especially aware of subtle, unexplained decreases in vision and/or visual field depression.

Hydroxychloroquine screenings are commonly performed in the clinic. However, actual findings of toxicity are rare. Because of this, subtle findings or early signs of toxicity may be overlooked. It is important for the provider to recognize that just because a condition is rare, it does not mean that they would not encounter a patient with it. We hope to increase awareness of subtle findings in hydroxychloroquine screening and emphasize the importance of thoroughly investigating for signs of toxicity, despite its rarity.

## References

[1] (2023). Hydroxychloroquine (Plaquenil). https://www.rheumatology.org/patients/hydroxychloroquine-plaquenil.

[2] Ponticelli C, Moroni G (2017). Hydroxychloroquine in systemic lupus erythematosus (SLE). Expert Opin Drug Saf.

[3] Danza Á, Graña D, Goñi M, Vargas A, Ruiz-Irastorza G (2016). [Hydroxychloroquine for autoimmune diseases]. Rev Med Chil.

[4] Yusuf IH, Charbel Issa P, Ahn SJ (2023). Hydroxychloroquine-induced retinal toxicity. Front Pharmacol.

[5] Plaquenil Hydroxychloroquine Sulfate Tablets USP (2024). Plaquenil hydroxychloroquine sulfate tablets USP. https://www.accessdata.fda.gov/drugsatfda_docs/label/2017/009768s037s045s047lbl.pdf.

[6] Marmor MF, Kellner U, Lai TY, Melles RB, Mieler WF (2016). Recommendations on screening for chloroquine and hydroxychloroquine retinopathy (2016 revision). Ophthalmology.

[7] Marmor MF, Chien FY, Johnson MW (2013). Value of red targets and pattern deviation plots in visual field screening for hydroxychloroquine retinopathy. JAMA Ophthalmol.

[8] Demeritt M, Reynolds S, Shechtman D, Davidson J (2024). How to succeed in plaquenil screenings. Review of optometry. https://www.reviewofoptometry.com/article/how-to-succeed-in-plaquenil-screenings.

